# Conceptual framework for the health and well-being of caregivers of HIV/AIDS orphans in North West Province, South Africa

**DOI:** 10.1186/s12912-024-02411-z

**Published:** 2024-10-14

**Authors:** Boitumelo Joy Molato, Salaminah Moloko-Phiri, Magdalena Koen, Molekodi Matsipane

**Affiliations:** NuMIQ Research Focus Area, School of Nursing, Faculty of Health Sciences, North West University Mahikeng campus, Private Bag X2046, Mmabatho, Mafikeng, 2745 South Africa

**Keywords:** Conceptual framework, Caregivers, HIV/AIDS orphans, Health, Well-being

## Abstract

**Background:**

The human immunodeficiency virus (HIV) and acquired immunodeficiency syndrome (AIDS) is an epidemic that continues to increase the burden of care among caregivers of HIV/AIDS orphans. Research has confirmed that HIV/AIDS orphans’ caregivers perform their duties in an unconducive environment. Consequently, this negatively impacts their health leading to inability to discharge caregiving duties effectively. After carefully considering the caregivers’ predicament, the researchers found it imperative to develop a conceptual framework for the North West Province as this province lacks a conceptual framework that addresses the health and well-being of caregivers for orphaned children.

**Methods:**

An exploratory, descriptive and contextual design was used in the study. The population for this study were HIV/AIDS orphans’ caregivers and ward-based professional nurses who also served as outreach team leaders. Non-probability purposive sampling technique was used to select participants for this study. Data was collected using individual semi-structured interviews, focus groups, and field notes. Six steps of thematic analysis were adopted to analyze collected data. The practice-orientated theory by Dickoff, James, and Wiedenbach guided the development of the conceptual framework. These six steps include the agent, recipient, context, procedure, dynamics, and terminus.

**Results:**

The study findings include home visits, health education, support during disclosure, routine monitoring of blood and growth, mobilization of support systems and resources, and utilization of government services.

**Conclusion:**

The conceptual framework seeks to improve the health and well-being of HIV/AIDS orphans caregivers so that they may provide high-quality care to the orphans. The framework guides outreach team leaders and nurses registered in primary health care institutions on the procedure to follow to improve and preserve the health of caregivers of children orphaned by HIV/AIDS.

## Background

The South African government initiated the primary healthcare re-engineering (PHC) effort in 2010 to initiate early disease detection, disease prevention, and health promotion [1]. This approach consists of three essential components: school health teams, district clinical specialist teams, and ward-based primary health care outreach teams (WBPHCOT) [1]. The WBPHCOTs comprise community health workers (CHWs) who get oversight, referral, and support from professional nurses. These professional nurses are known as outreach team leaders (OTLs) and are connected to nearby primary health care (PHC) institutions [14]. The acronym OTL will be used to refer to professional nurses.

One OTL leads a team of six CHWs in a municipal ward with the assistance of an environmental health officer and a health promoter [2]. The OTL ensures that the WBPHCOT’s work adheres to service delivery goals and that team members receive necessary guidance and assistance to fulfil these goals [3]. The OTLs account to the operations manager of the PHC facility that the WBPHCOT is registered under. When it comes to CHWs, they serve as the community’s initial point of contact for a variety of social and health services. They assist members of the community to make informed decisions about their health and psychological well-being. Additionally, they offer continuous care and support to people and families who are at risk because of long-term diseases and impoverished living conditions [3]. Research from several states globally indicates that having access to home and community-based health services in conjunction with care in permanent primary health care facilities is essential for achieving positive health outcomes [4]. Preventive and promotional health in rural communities in South Africa is positively impacted by WBPHCOT. Moreover, the initiative has improved PHC facility metrics to a greater extent [4]. Regardless of the strides demonstrated by WBPHCOT, existing literature emphasizes that the approach still has some challenges that interfere with its ability to achieve its objectives [Mhlongo et al., 2021]. Some of the challenges noted by the Mpumalanga qualitative study are excessive workload, meagre salaries and erratic payments [5]. The challenges identified by literature may have a negative impact on the effectiveness of the WBPHCOT. As a result, this may deprive vulnerable community members such as caregivers of HIV/AIDS orphans WBPHCOT services. Under normal circumstances, caregivers of HIV/AIDS orphans require multi-dimensional support such as social, emotional, physical, psychological, financial, and spiritual to effectively discharge caregiving duties [6]. The WBPHCOT are capable of providing such support services to the caregivers of HIV/AIDS orphans.

In some cases, the stressful caring environment that caregivers are exposed to during their care work affects their health and well-being. A study carried out in South Africa’s North West Province (NWP) revealed that unfavorable conditions in which they must discharge caregiving duties and the burden of caring for HIV/AIDS orphans adversely affected the health and well-being of HIV/AIDS orphans’ caregivers [7]. Health and well-being are two dimensions of health that are interrelated and influence each other. Activities promoting health help people transition from an unhealthy lifestyle to one that improves their health and well-being [8]. Improving the health and well-being of caregivers of HIV/AIDS orphans requires a systematic and scientific conceptual framework. The conceptual framework helps the stakeholders to understand connections between concepts or variables about the actual world [9–11]. Furthermore, it connects concepts, empirical research, and pertinent theories [12]. This study will use the developed conceptual framework to address health and well-being issues of HIV/AIDS orphans’ caregivers. Despite the problems reported by literature in the North West Province of South Africa about caregivers of HIV/AIDS orphans, there is still no conceptual framework to address the challenges. Based on the absence of a conceptual framework, the researchers identified a need to develop one that would address health and well-being challenges facing the caregivers of HIV/AIDS orphans in the North West Province, South Africa. Conceptual frameworks developed by [13, 14] followed the Practice-Oriented Theory (POT) by Dickoff, James and Wiedenbach to develop conceptual frameworks for psychosocial care and support for orphans and vulnerable children and for rape survivors diagnosed with post-traumatic stress disorders [13, 14].

Similarly, the researchers adopted POT survey list to develop a conceptual framework for health and well-being of caregivers of HIV/AIDS orphans. This article is part of the main study that aims to develop health promotion strategies to improve the health and well-being of caregivers of HIV/AIDS orphans in the Ngaka Modiri Molema district, North West Province, South Africa. The proposed conceptual framework is envisaged to inform the development of health promotion strategies for the health and well-being of the caregivers of HIV/AIDS orphans in the North West Province.

### Conceptual framework that guided the study

The POT survey list by [15] was adopted to develop a conceptual framework for the health and well-being of caregivers of HIV/AIDS orphans. The features of POT include the following:

- Agent (Who is the agent of the framework?

The term ‘agent’ refers to individuals who ensure the implementation of the proposed conceptual frameworks [15]. In addition, the agent is an individual within the structure who carries out tasks that are integrated into it.

- Recipient (who is the recipient of the framework).

The recipient refers to the person who benefits from the health services provided by the agent [15].

- Context (in which context will the framework be implemented).

The context refers to the environment or surroundings in which the activity will take place [15].

- Procedure (How will the framework be implemented).

The procedure refers to the process that is described in this framework that the agent followed to achieve the objectives of the study [15].

- Dynamics (What are the dynamics of the framework).

The dynamics refer to the actions taken by the agents to ensure that the process of the conceptual framework is followed and well implemented [15].

- Terminus (What will be the endpoint of the framework).

The concept “terminus” describes the conclusion or result of the conceptual framework [15]. The authors further explained the endpoint as the result of the agent’s efforts leading to improved outcomes.

### Method

#### Study design

The exploratory descriptive contextual design was used for this study. Qualitative research approach methods were used to perform three steps of the empirical phase. Phase 1 of this study had two objectives and objective one explored and described challenges faced by caregivers of HIV/AIDS orphans whilst objective two explored and described the coping mechanisms used by caregivers of HIV/AIDS orphans. Phase two explored support provided by OTLs to the caregivers of HIV/AIDS orphans. Exploratory research design was used to explore research questions for this study as they were not investigated before [16]. The descriptive research design was used to describe a phenomenon and its characteristics [17]. In terms of contextual research design, semi-structured interviews were conducted at the participants’ residences [18].

### Context

The study included five municipalities of the Ngaka Modiri Molema District, in the North West Province of South Africa. The municipalities where data were collected include Mahikeng, Ramotshere-Moiloa, Ditsobotla, Ratlou, and Tswaing. Ngaka Modiri Molema District occupies an approximate area of 28 114 km² [19]. Currently, Ngaka Modiri Molema District Municipality is home to 268 099 houses and 961 960 residents. Using the upper poverty line criterion, there are 640 000 (67.66%) persons living in poverty in Ngaka Modiri Molema District Municipality. This is 3.14% more than the 621 000 in 2008 [19].

### Population and sampling

The population were caregivers of HIV/AIDS orphans and OTLs. Purposive sampling was used to select both caregivers of HIV/AIDS orphans and the professional nurses. Eligibility to participate in the study was determined by the following factors: caregivers above 18 years of age, both male and female genders, have experience of caring for HIV/AIDS orphans. However, only female caregivers participated in this study. For OTLs, the criteria were that they should be registered with the South African Nursing Council (SANC) as Registered/Professional nurses, with/without additional qualification of PHC and have experience of more than a year working with the community, and be designated to PHC Re-engineering program in any of the five municipalities of Ngaka Modiri Molema district.

### Data collection

Three data collection methods namely semi-structured individual interviews, field notes, and focus group discussions. Face to face semi-structured individual interviews were used to explore and describe the challenges faced by caregivers of HIV/AIDS orphans. The interview guides for both interviews were designed by the researcher and moderated by the study leaders. The aim and objectives were used as guide in the development of the interview questions. The research questions for semi-structured individual interviews were: What are the challenges that you face when caring for HIV/AIDS orphans and what are the coping mechanisms that you use to care for HIV/AIDS orphans. In terms of focus groups, the objective was to explore and describe the support provided by OTLs to HIV/AIDS orphans’ caregivers. This phase had only one central question which was: what support do you provide to the caregivers of children orphaned by HIV/AIDS.

Field notes were used on both semi-structured individual and focus group interviews. The results gathered in phase 1 were combined in phase 2 to develop a conceptual framework for the health and well-being of caregivers of children orphaned by HIV/AIDS in the North West Province, South Africa. Data were collected during the day in both interviews. An audio recorder was used to record all the interviews after permission had been obtained from the participants. The duration for interviews were 45–60 min. Setswana language was used to collect data during semi-structured individual interviews which was later translated into English language before transcribing. The interviews were transcribed by the researcher and were later referred to a language expert for translation. Semi-structured focus group interviews were conducted using English language.

### Data analysis

Six steps of thematic data analysis were followed to analyze data collected in both phases 1 and 2 of data collection [20]. During the first step, the researchers and co-coder familiarized themselves with the data before beginning analysis. This was followed by a consensus meeting prior to finalising themes. In the second step, the research question served as a guide throughout the entire coding process. With regards the third step, the codes were grouped into themes to answer the research question and fulfil the objectives of the study [20, 21]. In respect of the fourth step, the themes were examined, pertinent data were categorized with each theme. The establishment of the relationship between themes and sub-themes was done in the fifth step [20, 21]. The sixth step was used for writing up and the existing literature was used to support the findings of the study [20]. Data analysis was done simultaneously with data collection to establish whether data saturation had been reached [22]. Field notes and all the data collected during Phase 1 were first examined independently, and then combined.

### Recruitment of participants

Pamphlets were emailed to the district communication officer to invite nurses to participate in the study. The subdistrict managers of the five local municipalities under Ngaka Modiri Molema district and the Primary Health Care (PHC) programme manager were also copied. The communication officers posted pamphlets on the Department notice boards. Nurses who showed interest in participating in the study called the research assistant to make an appointment with him for further details. The research assistant had a short interview with nurses who showed interest to assess their eligibility to participate in the study. The same procedure was followed in recruiting caregivers for children orphaned by HIV/AIDS. In their case, brochures were posted in public spaces such as clinics, stores, and churches. Interested caregivers also called the research assistant to make appointments to discuss where to meet and plan for data collection.

### Ethical consideration

This study was approved by North-West University Health Research Ethics Committee (NWUHREC), an institution of higher learning in the North West Province). The Ethics number for this study is NWU-00196-21-A1. Permission to conduct the study was obtained from North West Department of Health Ethics committee. This work is part of a larger scope which aims to develop health promotion strategies to improve the health and well-being of caregivers of children orphaned by HIV/AIDS. Written informed consent was obtained prior to the commencement of the study. The participants were informed about the purpose of the study and their rights prior participating in the study. Moreover, the participants were informed about the principle of autonomy which is voluntary participation. Thus, means that all participants were provided with complete information about the study and decided on their own whether to enroll [23]. Furthermore, the right to withdraw at any stage of the study was also explained to the participants. Privacy and confidentiality were maintained throughout the entire process of interviews [24]. Audio recordings and other documents such as informed consent were kept safe in a password protected cupboard that only study leaders and the researcher had access to.

### Trustworthiness

The following four trustworthiness principles credibility, dependability, confirmability, and transferability were adhered to. Firstly, credibility was maintained through prolonged engagement throughout the study. Interviews were recorded and field notes were taken to annotate the verbal and non-verbal cues. Participants in the study were consulted after data analysis for validation purposes [25]. Qualitative exploratory, descriptive, and contextual design methods were used in this study to ensure dependability. This principle was adhered to by ensuring detailed data transcription, data coding, data analysis, and the use of literature control [25]. Dependability principle was adhered to through independent data analysis by both the researcher and the coder. After analysing data independently, the two parties compared both themes and subthemes emerging from the study. Open-ended questions were applied, followed by probing for clarity. The existing literature was used to confirm or disprove the findings of the study [25]. Lastly, transferability was maintained by giving readers thorough contextual information to evaluate the findings’ applicability, a thick description of the data was preserved [25].

## Results

The results of semi-structured individual and focus group interviews were used to design the conceptual framework for the caregivers of HIV/AIDS orphans in the North West province as given in Tables 1, 2 and 3 below. Caregivers of HIV/AIDS orphans who gave consent to participate in the study were thirteen (13), their ages were ranging from 28 to 69. Among the caregivers of HIV/AIDS orphans, nine were unemployed whilst four of them were pensioners. All HIV/AIDS orphans’ caregivers who participated in the study were females. With regards professional nurses, twenty-seven (27) participated in the study twenty-three (23) of which were females and four (4) males. The four categories of age for the professional nurses were as follows: 30–39 were 2, 40–49 were 5, 50–59 were 12, and 60–69 were 08.

**Table 1:** Semi-structured individual interviews for objective 1: challenges faced by caregivers of HIV/AIDS orphans [Molato et al., 2024a]

Themes	Sub-themes
1. Lack of support for caregivers of HIV/AIDS orphans	1.1. Lack of family support. 1.2. Inadequate social support 1.3. Lack of financial support
2. Behavioural problems of HIV/AIDS orphans	2.1 Delinquent behaviour of HIV/AIDS orphans 2.2 Non-adherence to ART by HIV/AIDS orphans
3. Psychosocial distress of the caregivers	3.1 Stigma and discrimination of caregivers of HIV/AIDS orphans 3.2 Decreased ability to cope due to burden of caring
4. Negative health outcomes	4.1 Poor health

**Table 2:** Semi-structured individual interviews for objective 2: coping mechanisms used by caregivers [Molato et al., 2024b]

Main themes	Sub-themes
1. Support from significant others	1.1 Family support
	1.2 Neighbour support
	1.3 Life-partner support
2. Religious practices	2.1 Singing gospel songs
	2.2 Using prayer to cope
3. Social support services	3.1 Government support
	3.2 Support from local schools
	3.3 Stokvels and social clubs

**Table 3:** Focus group semi-structured interviews for objective 3: support provided by outreach team leaders to the caregivers [Molato et al., 2024c]

Main themes	Sub-themes
1. Conduction of home visits to the caregivers of HIV/AIDS orphans.	1.1 Promotion of medication adherence among children orphaned by HIV/AIDS. 1.2 Adoption of a Child Strategy. 1.3 Performance of routine blood and growth monitoring of children orphaned by HIV/AIDS. 1.4 Provision of physical support to the caregivers of children orphaned by HIV/AIDS during disclosure.
2. Coordination of multidisciplinary team support.	2.1 Referral to psychologists 2.2 Referral to social workers 2.3 Referral to the Dieticians/Nutritionist for nutritional support. 2.4 Referral to the law enforcement officers for security support.
3. Facilitation of support groups for both caregivers and children orphaned by HIV/AIDS.	3.1 Adherence clubs. 3.2 Provision of adolescent-friendly services.

## Discussion

This study aimed to develop a conceptual framework to improve the health and well-being of caregivers of children orphaned by HIV/AIDS. The focus of this section will be on the process followed to develop the conceptual framework (see the diagram below for more information).

### The relevance of the conceptual framework

The conceptual framework developed was clear and concise and set out clear six steps to improve the health and well-being of caregivers of HIV/AIDS orphans in the Ngaka Modiri Molema district, North West, South Africa. The conceptual framework has been developed for nurses, is user-friendly and can be used in all PHC service contexts. The information provided in the conceptual framework can also be used by policymakers to improve the health and well-being of caregivers of HIV/AIDS-affected children at national level.

### The assumptions of the conceptual framework

The main themes and subthemes arising from the empirical phase were combined to develop this conceptual framework. Dickoff et al. answered six crucial questions. The description of the practical orientation theory [15] is as follows:

Agent: (Who Is the Agent? )

In this study, agent refer to all professional nurses who work in public healthcare facilities and for WBPHCOTs as OTLs. The roles and responsibilities of OTLs as agents are to provide community outreach services, preventive, promotional, therapeutic, rehabilitation, and relief medicine, as well as primary health care to families and households [26]. Furthermore, OTLs communicate with communities to identify issues that affect their health and well-being and take the necessary measures to address identified health problems [27]. In the context of this study, agents must follow six steps of the conceptual framework developed to improve the health and well-being of HIV/AIDS orphans’ caregivers.

Recipient (Who is the recipient? ).

In this study, all recipients were caregivers of HIV/AIDS orphans. The caregivers refer to aunt, uncle, grandparents, sisters, brothers, over the age of 18, single or married, and taking care of children orphaned by HIV/AIDS in North West Province, South Africa. Caring for HIV/AIDS orphans in a stressful environment has negatively affected the health and well-being of caregivers. Consequently, this has decreased caregiver motivation to discharge their caring duties. Accordingly, caregivers may benefit from the conceptual framework if fully implemented as it aims to improve their health and well-being.

Context (In what context is the conceptual framework performed? ).

In this study, context refers to the homes of the caregivers of HIV/AIDS orphans where the conceptual framework will be implemented. The second context is the existing PHC facilities whereby OTLs report their operational activities. Each WBPHCOT in South Africa is connected to a PHC facility and comprises six or more community health workers (CHWs) in addition to an OTLs [28]. Additionally, a study conducted in South Africa reported that WBPHCOT acts as a liaison between the community and PHC facilities, they are eyes and ears, and hands of the profession [29].

The authors confirm that WBPHCOTs serve as the PHC facilities’ professional staff’s hands, eyes, and ears. The WBPHCOT is essential in helping PHC facility professional nurses connect with patients in the community in tracking the course of their illness [29]. If caregivers of children orphaned by HIV/AIDS consult at the PHC facilities, professional nurses should use the developed conceptual framework to address their health needs.

Procedure (How will the conceptual framework be implemented? ).

In this study, the procedure was developed based on the research findings and denotes the steps that will be followed by OTLs as agents when implementing the conceptual framework for caregivers of HIV/ADS orphans to achieve the terminus which is optimal health and wellbeing of caregivers of HIV/AIDS orphans. Home visits are vital as they help the OTLs in evaluating the living conditions and health practices of caregivers. According to a descriptive cross-sectional study carried out in Ghana, community health nurses use home visits as a medical platform to offer basic curative and preventative healthcare services to community members in the comfort of their own homes [30].

The authors further assert that community health nurses provide support to families in maintaining a healthy lifestyle and provide extra care to the most vulnerable members of the community [30]. In the context of this study, home visits should begin with the CHWs who should identify the health needs of the caregivers of children orphaned by HIV/AIDS. After identifying health problems experienced by caregivers of children orphaned by HIV/AIDS, the CHWs refer the matter to the OTLs for intervention.

Health education is a structured approach used by healthcare professionals to provide society with resources it needs to practice preventative care [31]. To improve community well-being, health education is essential because it addresses a variety of health issues, from social, psychological, mental health to chronic diseases, and it promotes healthy behaviours and knowledge across all age groups [32]. Otherwise stated, nurses need to be prepared to use health education as an instrument to modify their lifestyles for betterment of health [33]. Nurses must teach patients how to prevent illness, enhance their health, take prescribed medications, and make sure their efforts to return to normal function are successful [33]. Consequently, OTLs should ensure that caregivers of children orphaned by HIV/AIDS receive ongoing health education.

Routine blood and growth monitoring must be used as a tool to measure target achievement to keep an eye on whether caregivers are administering medication appropriately. Healthcare professionals in the South African context are empowered by policies, guidelines, and standards set by the Department of Health to monitor the growth of the children who are infected and by HIV/AIDS [34]. The World Health Organization (WHO) promoted viral load measurement because, in the wake of increased adherence support, it aids in distinguishing between treatment failure and non-adherence among people living with HIV (PLWHIV) [35].

Moreover, viral load testing provides PLWHIV with a gauge of their knowledge, control, and incentive to follow their treatment plan and comprehend the extent of their HIV infection [34]. According to descriptive research carried out in Mumbai, India, routine viral load testing prevents needless switching and allows for the early identification of treatment failures and referrals to second- and third-line regimens [36]. Moreover, a qualitative research conducted in Polokwane, South Africa, affirms that the program assists in identifying developmental milestones and growth failing in children so that prompt interventions can be implemented [37]. This study suggests that OTLs must not strictly rely on guidelines, protocols, and policies of HIV/AIDS management in South Africa. Flexibility is needed in the cases of implementation of the standards outlined by Department of Health to manage HIV/AIDS in South Africa. For instance, if children living with HIV (CLWHIV) present with failure to thrive (FTT), the OTLs must not wait for the period prescribed to monitor blood, it should be attended to immediately. Growth of CLWHIV should be monitored regularly during home visits or consultation at the PHC facilities.

The OTLs must provide both caregivers and HIV/AIDS orphans with emotional support during disclosure. Previous research highlighted that there are caregivers of children orphaned by HIV/AIDS who struggle to carry out disclosure process. Governments should set up mechanisms to capacitate caregivers with knowledge and skills that boost their confidence [38]. A narrative review by Molato et al. [39] highlights the following elements as having an impact on HIV status disclosure to affected children: the children’s age; the primary caregivers’ educational status; stigma and discrimination; and a lack of knowledge and skills about HIV status disclosure.

A qualitative exploratory descriptive study carried out in the Limpopo Province, South African caregivers for CLWHIV were not trained in initiating HIV disclosure [40]. As a result, caregivers of HIV/AIDS orphans who lack the ability to disclose believed that healthcare professionals should help them to conduct disclosure process [41]. Despite inadequate support, the findings of the study conducted by Muditambi et al. [40]. revealed that caregivers had been assisted by the healthcare practitioner on how to disclose HIV status to CLWHIV.

OTLs must initially explore the effective coping mechanisms that caregivers use to cope with the burden of caring for children orphaned by HIV/AIDS. In line with existing literature, the qualitative study conducted in the North West Province highlighted that caregivers who relied on religious practices managed to cope with all the difficulties that interfered with their ability to discharge effective and efficient care for HIV HIV/AIDS orphans [6]. According to the authors, some of the caregivers used singing of gospel hymns and prayer to cope whenever they found themselves in a predicament aggravated by execution of caring tasks among children orphaned by HIV/AIDS. Similar results were reported by the study conducted in Ethiopia where caregivers put God first in everything that they were doing and when experiencing difficulties that confronted them in caring for orphaned children [42].

They hoped that through God everything was possible, He couldnot leave his children to suffer without providing solutions to their problems [42]. The findings of this study, together with those from earlier research, demonstrate the necessity of having trust and hope in God and the necessity of involving Him in whatever we do. The study conducted by Howard et al. [43] affirms that spirituality improves mental and physical health, resilience and they are significantly associated with higher life satisfaction. As Christians, we affirm that God is willing to listen to our suffering and provides interventions to help us overcome the obstacles we encounter. It is the responsibility of OTLs to ensure that all children who have lost parents due to HIV/AIDS receive the monthly governmental foster care grants (FCG). Social grants play an important role in the children’s lives as they help them in accessing essentials such as food, stationery, and school uniform. The welfare and well-being of the orphaned children depend heavily on the state providing the FCG to alleviate the financial strain on their families [44]. The FCG program also gives foster parents the tools and assistance they need to ensure that the needs of orphaned children are appropriately met [44]. It is thus imperative that during home visits OTLs screen for caregivers who do not receive FCG, establish the root cause and intervene.

OTLs should mobilize support networks for caregivers of children orphaned by HIV/AIDS. Research has indicated that social support from friends, family, and other social networks improves life quality and serves as a crucial defense against mental health issues [45]. This was evident in the study conducted in the NWP in which caregivers of HIV/AIDS orphans relied on the support of significant others to cope with all challenges that interfered with their ability to carry out their day-to-day activities [6]. OTLs must also motivate caregivers to join social clubs and groups. According to literature, caregivers who joined social clubs and groups were able to learn more about HIV care, lessen discrimination and stigma, encourage disclosure, and improve their relationship with orphaned children [46]. Evidently, caregivers should be advised and encouraged to join social clubs.

Social connections are usually made between people who have common social interests rather than between strangers [47]. As a result, while examining how social networks arise, it is also important to carefully examine the social environment, in particular the quantity and variety of social clubs [47]. Common examples of social clubs include support groups, stokvels, family society, and sports clubs. Considering this, OTLs must counsel caregivers to become members of social clubs like stokvels, which are recognized in literature as the primary means of reducing poverty and promoting social mobility [48]. The authors further claimed that members of stokvels can meet their fundamental necessities. Members of stokvels could invest, save, and eventually amass assets [48]. Previous studies reported that stokvels helped caregivers save a lump sum of money that they shared at the end of the year to buy bulk groceries [6, 49]. According to the authors, caregivers were relieved that they didn’t have to buy groceries after receiving social grants because the groceries lasted for almost three months.

Caregivers had to reallocate the social grant funds for other essentials purchases. As far as mobilization of the resources is concerned, OTLs must ensure that community resources such as multi-purpose skills centers are utilized effectively to empower caregivers of children orphaned by HIV/AIDS with knowledge and skills to sustain their life. In support of these findings, a cohort longitudinal study conducted in Swaziland revealed that caregivers of children orphaned by HIV/AIDS were capacitated with skills to start businesses [50].

According to the authors, among the Women of Recreation, Tourism & Hospitality (WORTH) members who were caregivers for orphaned children, one-third (33.4%) were able to start a business, and 41% of the caregivers were able to grow their businesses from one to two (or more) [50]. Literature about measures put in place by government or non-governmental organizations (NGOs) to develop caregivers of children orphaned by HIV/AIDS remain unknown in the South African context. Therefore, it is essential for OTLs to work with various stakeholders who are interested in community development to empower HIV/AIDS orphans’ caregivers. It should be the prerogative of the OTLs to ensure that all children who have lost parents due to HIV/AIDS receive monthly foster care grants from the government.

Social grants play a significant role in their lives as they assist them in accessing essentials such as food, stationery, and school uniform. According to a qualitative study conducted in South Africa; social grants given by the government to HIV/AIDS orphans’ caregivers are insufficient to meet their daily needs [51]. The authors further stated that caregivers had respectfully asked for an increase in foster care grant to cover the costs of various extras, such as educational policies, which are crucial to ensuring that orphaned children have a respectable future.

Dynamics (What are the dynamics of the conceptual framework).

In this study, dynamics refer to the energy source that will drive the activity and it could be chemical, physical, biological, mechanical, emotional, spiritual, psychological, and resilience. OTLs lead teams and work closely with CHWs by supervising them to ensure that professional duties are carried out accordingly. Therefore, it is important that agents of the framework induct CHWs on the conceptual framework so that they can be aware of their roles and responsibilities.

As custodians of the provision of healthcare services in South Africa, the Department of Health outlined the responsibilities of OTL to include collaborating with non-healthcare professionals, private health centres, and non-governmental organizations to eradicate community health-related issues. This research confirms the findings of previous studies that emphasise the central role of MDT as a means of assembling a team of healthcare professionals from various specialities to design individualized plans for the treatment of patients [52]. The MDT stakeholders reported in this study include nurses, social workers, psychologists, dieticians, and many others who fall in the medical field.

Caregivers who are encountering psychosocial challenges should be referred to the social workers. This procedure aligns with the recommendation of a qualitative study carried out in South Africa, which suggested that foster parents of abandoned children exhibiting social and welfare issues ought to be referred to the social workers [53]. The caregivers who are not coping due to emotional and psychological distress should be referred to the psychologists. Previous studies have emphasized that psychologists are responsible for addressing social issues and advancing the health and well-being of community members [54]. They also work to create preventative initiatives and educate the public.

Dietitians must be involved by the OTLs to start community nutrition, which is the act of assisting people and groups to create wholesome eating habits to promote wellbeing and fend against disease [55]. To improve the health and nutrition status of people living with HIV, the expertise of a registered dietitian/nutritionist as part of the health care team should be considered. Previous research has demonstrated that nutrition education or counselling improves dietary intake and nutritional status in adults living with HIV/AIDS significantly [56–59]. The authors stressed that to enhance the health and nutritional condition of those living with HIV, dietitians and nutritionists should be considered. OTLs can also facilitate collaboration with non-healthcare professionals to advance the needs of caregivers of children orphaned by HIV/AIDS who are socially dysfunctional.

OTLs as the framework’s agents have a responsibility to maintain communication between MDT and other stakeholders. There should be constant communication between the agent and MDT to facilitate the health and well-being of caregivers. Patient safety may be at risk when members of the healthcare team are not communicating properly [60]. As per the authors’ assertion, inadequate communication exacerbates the likelihood of medical errors [60]. Therefore, effective communication is the primary factor influencing the team’s performance and quality [61]. The author went on to explain that effective communication forms the cornerstone of all subsequent endeavours and guarantees favourable patient outcomes [61].

Health promotion has been the cornerstone of the Healthy People project, which aims to promote “health and well-being” more broadly [62]. Currently, health promotion process contributes to both individual and societal well-being in addition to health [62]. In terms of health promotion, illness prevention, treatment, and care, nurses are the backbone of health teams, they play a significant role in ensuring that the goals of the program are achieved [63]. In the framework of this study, OTLs are required to plan, coordinate, and carry out health promotion activities to improve the health and wellbeing of HIV/AIDS orphans’ caregivers in the North West Province. Moreover, OTLs should strengthen community action for health, build their skills, and create supportive environments for health to meet the goal of improving the health and well-being of the caregivers [64].

Terminus (What is the end point of the conceptual framework? ).

**Fig. 1:**
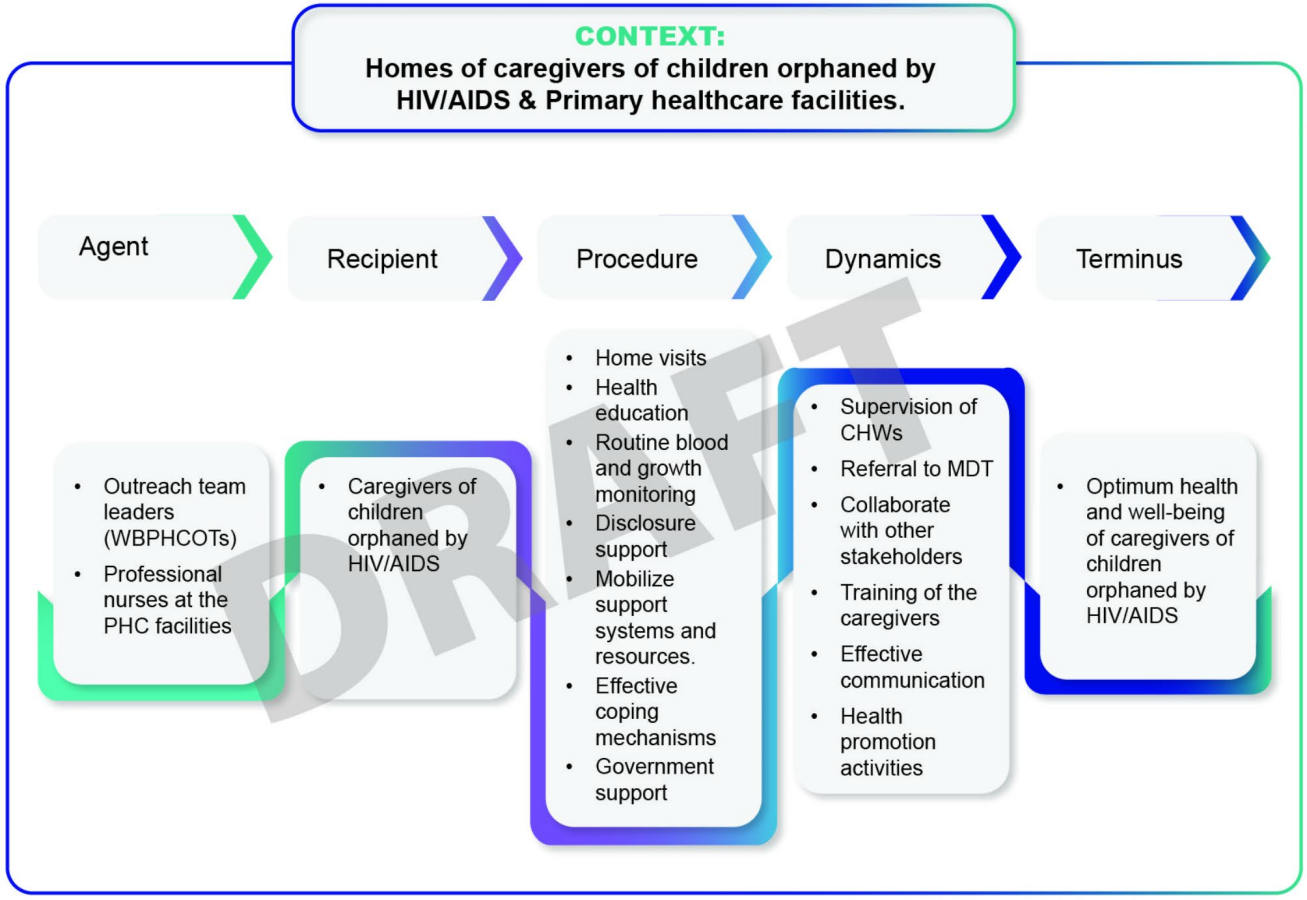
Conceptual framework

In this study, terminus refers to the endpoint of the conceptual framework. The agent must ensure that the procedure of the conceptual framework is carried out within the context of the developed framework. After successful implementation of the conceptual framework, caregivers of HIV/AIDS orphans should have optimum health, functional support system, effective coping mechanisms, and active execution of caregiving responsibilities (see the diagram illustrated in Fig. 1 below for more details).

### Limitations

The punctuality of the participants was a problem, the researcher had to start late, and other appointments scheduled for that day were affected by such incidents.

## Conclusions

This study developed a conceptual framework for health promotion strategies targeting HIV/AIDS orphans’ caregivers in the Ngaka Modiri Molema district. The framework addresses the unique challenges faced by caregivers and emphasizes proactive interventions to improve their well-being and the health of the children under their care. It has significant implications for policy and practice, providing guidance for the development of targeted interventions and collaboration among stakeholders. However, the study’s limitations should be acknowledged, including the need for further research to validate and refine the framework in different settings, and the reliance on qualitative data. Overall, the framework has the potential to positively impact caregivers’ lives and the health outcomes of the children they care for.

### Recommendations

The researchers suggest that training and unequivocal support should be provided to the caregivers to enable them to conduct disclosure processes independently. Alternatively, OTLs can also avail themselves during disclosure to provide both HIV/AIDS orphans and caregivers emotional support to alleviate distress. Additionally, OTLs should assist by being sympathetic to ease their discomfort. The significance of disclosure of HIV status to CLWHIV should be a key factor that drives both caregivers and OTLs. Moreover, caregivers of HIV/AIDS orphans who are not Christians should be motivated to consider it based on the positive outcomes reported in this study. The researchers further recommend more studies to explore the effectiveness of spirituality in improving the well-being of HIV/AIDS orphans’ caregivers.

## Data Availability

There will be no public sharing of any of the raw data, it will remain private and confidential. This includes audio recordings and interview transcripts.
