# Correction: Conceptual framework for the health and well-being of caregivers of HIV/AIDS orphans in North West Province, South Africa

**DOI:** 10.1186/s12912-025-02935-y

**Published:** 2025-03-11

**Authors:** Boitumelo Joy Molato, Salaminah Moloko-Phiri, Magdalena Koen, Molekodi Matsipane

**Affiliations:** NuMIQ Research Focus Area, School of Nursing, Faculty of Health Sciences, North West University, Mahikeng Campus, Private Bag X2046 Mmabatho 2745, Mafikeng, South Africa

**Correction: BMC Nurs 23, 754 (2024)**

10.1186/s12912-024-02411-z

Following the publication of the original article [1], the author group identified two errors. The description of the phases in the Data collection section was revised and the number and breakdown of study participants in the Results section was corrected as detailed below.

Error 1:

**Correct**

“The results gathered **in phase 1 were combined in phase 2** to develop a conceptual framework for the health and well-being of caregivers of children orphaned by HIV/AIDS in the North West Province, South Africa”.

**Incorrect**

“The results gathered **in phases 1 and 2 were combined in Phase 3** to develop a conceptual framework for health and well-being of caregivers of children orphaned by HIV/AIDS in the North West Province, South Africa”.

Error 2:

**Correct**

“With regards professional nurses, **twenty-seven (27)** participated in the study **twenty-three (23)** of which were females and four (4) males. The four categories of age for the professional nurses were as follows: 30–39 were 2, 40–49 were 5, 50–59 were 12, and 60–69 were **08**”.

**Incorrect**

“With regards professional nurses, **thirty-seven (37)** participated in the study **thirty-three (33)** of which were females and four (4) males. The four categories of age for the professional nurses were as follows: 30–39 were 2, 40–49 were 5, 50–59 were 12, and 60–69 were **18**”.

The original article [1] has been updated.
